# Presence of Androgen Receptor Variant in Neuronal Lipid Rafts

**DOI:** 10.1523/ENEURO.0109-17.2017

**Published:** 2017-08-29

**Authors:** Jo Garza-Contreras, Phong Duong, Brina D. Snyder, Derek A. Schreihofer, Rebecca L. Cunningham

**Affiliations:** Center for Alzheimer’s and Neurodegenerative Disease Research and Center for Neuroscience Discovery, Institute for Healthy Aging, University of North Texas Health Science Center, Fort Worth, TX 76107

**Keywords:** Androgen receptor, caveolin, G proteins, lipid rafts, membrane, signaling

## Abstract

Fast, nongenomic androgen actions have been described in various cell types, including neurons. However, the receptor mediating this cell membrane–initiated rapid signaling remains unknown. This study found a putative androgen receptor splice variant in a dopaminergic N27 cell line and in several brain regions (substantia nigra pars compacta, entorhinal cortex, and hippocampus) from gonadally intact and gonadectomized (young and middle-aged) male rats. This putative splice variant protein has a molecular weight of 45 kDa and lacks an N-terminal domain, indicating it is homologous to the human AR45 splice variant. Interestingly, AR45 was highly expressed in all brain regions examined. In dopaminergic neurons, AR45 is localized to plasma membrane lipid rafts, a microdomain involved in cellular signaling. Further, AR45 protein interacts with membrane-associated G proteins Gαq and Gαo. Neither age nor hormone levels altered AR45 expression in dopaminergic neurons. These results provide the first evidence of AR45 protein expression in the brain, specifically plasma membrane lipid rafts. AR45 presence in lipid rafts indicates that it may function as a membrane androgen receptor to mediate fast, nongenomic androgen actions.

## Significance Statement

Evidence has been building for the existence of a membrane androgen receptor, but the nature of this receptor remains elusive. We predicted that the membrane androgen receptor is a splice variant that is present in lipid raft microdomains within the neuronal plasma membrane. Indeed, an androgen receptor that is lacking an N-terminal domain and complexed to Gαq and Gαo G proteins was highly expressed in lipid rafts in dopaminergic neurons. This putative splice variant provides a potential target for mediating androgen’s nongenomic actions.

## Introduction

In addition to peripheral expression in many tissues, the androgen receptor (AR) is expressed in many areas of the brain, including brain regions not involved in regulating classic endocrine functions (e.g., substantia nigra, hippocampus, entorhinal cortex; McEwen, 1980; Song et al., 1991; Kerr et al., 1995; Kritzer, 1997; Kritzer and Creutz, 2008; Sarkey et al., 2008). The classic intracellular AR is involved in genomic transcription (Ozanne et al., 2000; Edwards and Bartlett, 2005; Smith and Toft, 2008; Green et al., 2012). This AR is composed of eight exons that code for a 110-kDa protein belonging to the steroid receptor superfamily of nuclear transcription factors (Claessens et al., 2008). The classic AR has four distinct regions: a regulatory NH_2_ terminal domain (NTD; Ferro et al., 2002; Ding et al., 2004, 2005; Callewaert et al., 2006; Claessens et al., 2008), a DNA binding domain (DBD), a hinge domain, and a ligand binding domain (LBD) extending to the C-terminal domain (CTD; Claessens et al., 2008).

Evidence for a nongenomic membrane AR (mAR) has been accumulating since the 1990s. Investigators have found that even cell-impermeable androgens can have fast cellular effects (Steinsapir et al., 1991; Gorczynska and Handelsman, 1995; Benten et al., 1999; Estrada et al., 2003, 2006; Holmes et al., 2013). Rapid membrane effects of androgens, via testosterone conjugated to bovine serum albumin (BSA), have been observed in neuronal cell lines (N27 and SH-SY5Y), resulting in an oscillatory pattern of intracellular calcium release that is unaffected by AR antagonists or knockdown of the classic intracellular AR (Estrada et al., 2006; Holmes et al., 2013). Unlike the classic AR, the mAR has not been cloned or purified. Different theories have proposed that the mAR could be the classic AR anchored to the plasma membrane (Pedram et al., 2007), an unknown AR (Hatzoglou et al., 2005), or even a splice variant of the classic AR (Foradori et al., 2008).

Numerous AR splice variants have been found outside the nervous system. These variants originate from alternative splicing at different promoters and thus increase AR complexity and biological functions (Modrek and Lee, 2002; Roberts and Smith, 2002; Stamm, 2002; Ahrens-Fath et al., 2005; Jagla et al., 2007; Hu et al., 2012). Most of these AR splice variants have a truncated LBD in the CTD, which can result in a constitutively active AR (Sun et al., 2010; Watson et al., 2010; Hu et al., 2012). In contrast to the loss of LBD, some of these AR splice variants exhibit partial to full loss of the NH_2_ regulatory domain. For example, both AR splice variants (AR8 and AR45) have truncated NTD (Jenster et al., 1991; Ikonen et al., 1998; Ahrens-Fath et al., 2005; Yang et al., 2011).

The AR splice variant, AR45, is the least understood variant and has been characterized only in humans. This splice variant lacks the entire NTD (exon 1). This deletion decreases the protein molecular weight of AR from 110 to 45 kDa (Ahrens-Fath et al., 2005). Functionally, in the periphery, AR45 binds androgens via LBD and translocates to the nucleus. It can homodimerize with other AR45 receptors or heterodimerize with classic AR. AR45 can act as a negative modulator of AR activity via competitive inhibition of AREs by homodimers or by interfering with coactivator recruitment necessary for AR activity (Jenster et al., 1991; Ikonen et al., 1998; Ahrens-Fath et al., 2005). AR45 is expressed in multiple tissues, such as muscle, lung, heart, breast, uterus, and prostate (Ahrens-Fath et al., 2005; Weiss et al., 2007; Wu et al., 2008). Although AR45 protein expression was not observed in total brain homogenate (Ahrens-Fath et al., 2005), a recent study found low mRNA expression of AR45 in human brain tissue that was commercially obtained from an aged population (Hu et al., 2014). Neuronal function of AR45 is unknown.

To determine whether the putative mAR could be AR45 in different brain regions (substantia nigra pars compacta, CA1 region of the hippocampus, and the second layer of the entorhinal cortex), we measured protein expression of AR using antibodies targeting the CTD and NTD of the AR. Because neuronal mAR has been associated with intracellular calcium signaling (Steinsapir et al., 1991; Gorczynska and Handelsman, 1995; Benten et al., 1999; Estrada et al., 2003, 2006; Holmes et al., 2013), we examined if the mAR complexed with G proteins.

## Materials and Methods

### Reagents

Testosterone (A6950-000) was obtained from Steraloids. Goat anti-rabbit (sc-2004), androgen receptor C-19 (sc-815), androgen receptor N-20 (sc-816), AR441 (sc-7305), AR N20P (sc-515856), AR C19P (sc-515863), Gαq (sc-393), Gαs (sc-823), Gαo (sc-393874), Gαi_1_ (sc-391), Gαi_2_ (sc-13534), and Gαi_3_ (sc-262) antibodies were obtained from Santa Cruz Biotechnologies. Flotillin (3253) and caveolin-1 (3267) antibodies were obtained from Cell Signaling Technology. Alexa Fluor 594 antibody was purchased from Jackson ImmunoResearch Laboratories. GAPDH (GT239) antibody was obtained from GeneTex. Biotinylated anti–rabbit IgG (BA-1000) was purchased from Vector Laboratories. Androgen R/NR3C4 (AB58761) was obtained from R&D Systems. DMSO was purchased from VWR. RPMI 1640, penicillin-streptomycin (PS), and trichloroacetic acid (BDH9310) were purchased from VWR. Fetal bovine serum (FBS) and PBS were obtained from Corning. Charcoal-stripped FBS (CS-FBS) was purchased from Atlanta Biologicals. Mounting medium was obtained from Vector Laboratories (H-1200). SuperSignal West Pico/Femto chemiluminescent substrates, dithiothreitol (DTT), and Clean Blot IP Detection Reagent (PI21230) were obtained from Thermo Fisher Scientific. Deoxycholic acid (D-6750), inactin (T133), horse serum (H1138), and Triton X-100 were purchased from Sigma-Aldrich. Tris, Any KD polyacrylamide gel, Tris-glycine buffer, and PVDF membranes were purchased from Bio-Rad. Total testosterone ELISA (RTC001R) was purchased from BioVendor. Testosterone was made using an ethanol vehicle (final concentration of ethanol <0.001%).

### Animals

Experiments were conducted on young adult (3 mo) or middle-aged retired breeder (9–12 mo) male Sprague-Dawley rats (Charles River). Animals were either gonadally intact or were gonadectomized to remove circulating gonadal hormones. Rats were individually housed in a temperature-controlled environment on a 12:12-h light-dark cycle. All rats had *ad libitum* access to food and water. Animals were weighed at the time of surgery and at death. All experimental procedures were approved by the University of North Texas Health Science Center IACUC in accordance with the guidelines of the Public Health Service, the American Physiologic Society, and the Society for Neuroscience for animal care and use.

### Gonadectomy

Under 2.5% isoflurane, a midline scrotal incision was made to expose the spermatic cord. The spermatic cord was tied off with sterile sutures, and the cord was cut distal to the thread to remove the testes. The incision was closed with sterile absorbable sutures (Cunningham et al., 2011).

### Micropunch tissue dissection

One week after surgery, each rat was anesthetized with 2.5% isoflurane and decapitated. The brain was removed from the skull, rinsed in ice-cold PBS, and placed into a brain matrix (Braintree Scientific) on ice. Using razor blades, the brain was cut into 1-mm coronal sections. The razor blades were placed on dry ice to freeze the freshly cut brain sections. Punches were obtained from the SN pars compacta (–5.30 mm from bregma), second layer of the entorhinal cortex (ETC; –5.30 mm from bregma), and the CA1 layer of the dorsal hippocampus (–4.52 mm from bregma) using 1-ml syringes with a 20-gauge blunt needle (Snyder et al., 2017). Samples were placed into microcentrifuge tubes, snap-frozen on dry ice, and stored at –80°C or immediately homogenized into whole-cell lysates.

### *In vitro* cell culture

The immortalized neuronal cell line 1RB3AN_27_ (N27), derived from fetal rat mesencephalic tissue, is positive for tyrosine hydroxylase expression (TH^+^; Clarkson et al., 1999; Anantharam et al., 2007; Carvour et al., 2008). N27 cells were cultured and maintained at 37°C in 5% CO_2_. Medium used was RPMI 1640 supplemented with 10% FBS and 1% PS. N27 cells were used only in passages 13–19. Before hormone treatment for whole-cell lysate experiments, the medium was switched to RPMI 1640 with CS-FBS to avoid confounding from the presence of hormones in the serum. Cells were exposed to testosterone (100 nM) or vehicle control for 18 h and collected for protein. The testosterone concentration used in this study was 100 nM, representing the high end of the normal testosterone range in men (Mooradian et al., 1987; Kelly et al., 1999; Smith et al., 2000; Zitzmann et al., 2002).

### Whole cell lysates

For *in vitro* preparations, N27 cells were plated in 100 × 20-mm plates at a density of 8.0 × 10^4^ cells per plate. After treatments, cells were washed with PBS and lysed using a cocktail of NP40 and phosphatase inhibitors (1:100) on ice. For *in vivo* preparations, brain region micropunches were incubated with RIPA homogenization buffer with DTT (1 µM), EDTA (1 mM), and protease and phosphatase inhibitors (1:200) for 30 min on ice, sonicated (QSonica) at 20% amplitude, and pulsed 3 times for 3 s. Next, lysates were centrifuged at 4°C for 20 min at 12,000 × *g*. Protein concentrations were determined using the BCA assay (Thermo Fisher Scientific) according to the manufacturer’s instructions.

### Detergent-free cellular fractionation and sucrose density analysis of membrane lipid rafts

**Figure 1.**
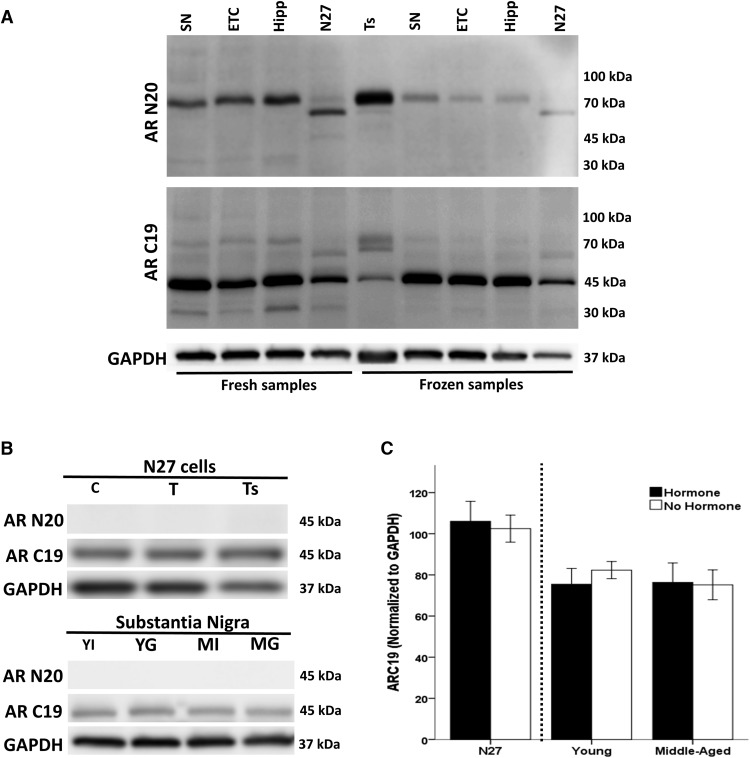
Androgen receptor protein expression. AR protein expression was quantified in N27 cells and SN, ETC, hippocampus, and testes micropunches from young gonadally intact male rats (*n* = 3). Antibodies targeting either the NTD (AR N20) or the CTD (AR C19) of the AR were used. Low protein expression of full-length AR (110 kDa) was found in all samples, but high protein expression of AR fragments (70 and 30 kDa) was observed in fresh samples and to a lesser extent in frozen samples. Protein expression at 45 kDa (AR45) was observed in all samples and was unaffected by temperature (***A***). To determine the role of testosterone and age on AR45 expression, different hormone groups were used: N27 cells treated with vehicle control (*n* = 8) or 100 nM testosterone (*n* = 8) and SN from young and middle-aged male rats that were either gonadectomized (*n* = 3) or gonadally intact (*n* = 3; ***B***, ***C***). Testosterone did not affect AR45 expression in N27 cells. Neither hormones nor age affected AR45 expression in SN (***B***, ***C***). C, control; T, testosterone; Ts, testes; YI, young gonadally intact male rats; YG, young GDX male rats; MI, middle-aged gonadally intact male rats; MG, middle-aged GDX male rats; SN, substantia nigra pars compacta; ETC, 2nd layer of the entorhinal cortex; Hipp, CA1 region of the dorsal hippocampus.

N27 cells were plated in 100 × 20-mm plates at a density of 8.0 × 10^4^ cells per plate. After treatments, cells were washed with PBS and lysed with a cocktail of hypotonic homogenization buffer and phosphatase inhibitors (1:100) on ice. Each sample (*n*) consisted of two 100 × 20-mm plates. For micropunches, a total of 0.025 g of SN tissue was homogenized in hypotonic homogenization using a sonicator to homogenize the tissue. After homogenization, cellular fractionation followed by sucrose density analysis of membrane lipid rafts was performed (Jeske et al., 2003, 2004). Cell lysate from either N27 cells or micropunches was centrifuged at 1000 × *g* for 5 min at 4°C to separate the nuclei. The supernatant was centrifuged at 16,000 × *g* for 30 min at 4°C to separate the cytosolic proteins from the mitochondria, Golgi fragments, and the plasma membrane. The pellet was then resuspended in homogenization buffer supplemented with 500 mM Na_2_CO_3_ (Song et al., 1996). The resuspended membrane pellet was placed into a sucrose flotation-gradient fraction using 5%/35%/45% discontinuous gradient that was spun at 175,000 × *g* for 18 h at 4°C in an Optima ultracentrifuge Model LE-80K (Beckman Coulter) using a swing bucket rotor (SW 50.1; Beckman Coulter). After the high-speed centrifugation, equal-volume fractions were taken from the top layer of the gradient, resulting in nine fractions (low-density proteins at the top gradient layers to high-density proteins at the bottom gradient layers). Protein was precipitated using the trichloroacetic acid (TCA) method (Link and LaBaer, 2011). The pellet was incubated in 0.15% deoxycholic acid and then 72% trichloroacetic acid, followed by 16,000 × *g* centrifugation for 30 min at room temperature. The pellet was resuspended in RIPA lysis buffer, Laemmli loading buffer, and 2 M Tris. The sample was loaded into polyacrylamide gels for electrophoresis and Western blot protein analysis. Lipid raft experiments were replicated three times. Only frozen samples were used to decrease AR fragment protein expression (Fig. 1*A*).

### Coimmunoprecipitation

Because C19^+^/N20^–^ AR protein expression at 45 kDa was observed only in the membrane fraction (not the cytosol or nuclear fractions), whole-cell lysates were used. Protein (25 μg) was incubated overnight at 4°C in a cocktail containing RIPA lysis buffer and 1 μg primary antibody (AR C-19 or Gαq). A Sepharose bead slurry was coupled to each sample by incubating at 4°C overnight. Samples were washed, eluted, and resuspended in 4× Laemmli buffer. To avoid IgG band interference, the blot was incubated with Clean Blot IP Detection Reagent HRP, per the manufacturer’s instructions. Coimmunoprecipitation experiments were replicated four times. Only frozen samples were used to decrease AR fragment protein expression (Fig. 1*A*).

### Western blot

Equal amounts (20 μg protein) of denatured whole cell lysates, micropunch tissues, cellular fractions, or lipid raft fractions were loaded into a Bio-Rad Any KD polyacrylamide gel, electrophoresed in Tris-glycine buffer, and transferred onto a PVDF membrane. Membranes were blocked for 30 min with 5% nonfat milk in TBS-Tween at room temperature. After blocking, membranes were incubated with specific primary antibodies (AR C-19 1:1000, AR N-20 1:750, AR441 1:1000, Androgen R/NR3C4 1:1000, GAPDH 1:10,000, Gαq 1:1000, Gαs 1:10,000, Gαi_1-3_ 1:10,000, Gαo 1:1000, flotillin 1:1000, and caveolin-1 1:10,000) in TBS-Tween with 1% nonfat milk for 2 h or overnight at 4°C. Afterward, the membranes were washed every 10 min for 30 min and incubated with secondary antibodies at 1:1000 in TBS-Tween with 1% nonfat milk for 30 min at room temperature. Protein bands on the membrane were visualized using an enhanced chemiluminescence detection assay (Thermo Fisher Scientific). Protein band intensities were imaged using GeneSys software corresponding with the G:Box Chemi XRQ system (Syngene). Protein band densities were quantified by NIH ImageJ densitometer software and normalized to GAPDH for whole-cell lysates, using the equation (mean gray value for protein of interest)/(mean gray value of GAPDH) × 100. All antibodies used in this study are commercially available. The specificity of the antibodies was assayed by using primary antibodies that were preabsorbed with blocking peptides (N20P 1:50, C19P 1:20) at 4°C overnight on representative blots. Previous immunohistochemical studies have shown specificity for AR-C19 and AR N-20 (Kritzer and Creutz, 2008; Holmes et al., 2016).

### Immunohistochemistry

One week after gonadectomy or sham surgery, each rat was anesthetized with Inactin (100 mg/kg, i.*p*.; Sigma), transcardially flushed with 0.1 M PBS (100–200 ml), and then perfused with 4% paraformaldehyde in 0.1 M PBS (300–500 ml; Gottlieb et al., 2006; Ji et al., 2007; Cunningham et al., 2011). The brain was removed from the skull and postfixed with 4% paraformaldehyde in 0.1 M PBS for 24 h. Brains were stored in vials containing 30% sucrose in PBS at 4°C for 3–4 d before sectioning. Brains were cut into 3 separate sets of 40-μm coronal sections using a cryostat (CryoStar NX70, Thermo Fisher Scientific). Coronal sections containing the SN (–4.80 to –6.04 mm from bregma) were blocked with 3% PBS diluent (3% horse serum in PBS with 0.25% Triton X-100) for 2 h at room temperature followed by overnight incubation at 4°C with primary antibody (AR C-19 or AR N-20 at 1:500) in 3% PBS diluent. Afterward, the sections were washed with PBS. Serial sections incubated with AR C-19 or AR N-20 antibodies were then incubated with secondary antibodies (Alexa Fluor donkey anti-goat 1:1000) at room temperature for 5 h (Nedungadi et al., 2012). Afterward, sections were washed with PBS. Sections were mounted on slides and sealed with mounting medium. After sealing, the slides were stored at 4°C. Images were captured from each section using an epifluorescent inverted microscope (VWR) equipped with a digital camera (Photometrics Cool Snap Myo; Nikon) and imaging software (NIS Elements, Br 4.50.00; Nikon).

### RT-PCR

RNA was extracted from tissue punches of substantia nigra, entorhinal cortex, hippocampus, and confluent N27 cells using a Qiagen RNeasy kit and quantified by spectrophotometry. One microgram of each sample was reverse transcribed using a High Capacity cDNA Reverse Transcription kit from Applied Biosystems. AR intron spanning primers (Table 1) were used for PCR in 100-ng equivalents from each RT reaction over 35 cycles with an annealing temperature of 56°C using 300 nM of each primer and GoTaq polymerase (Promega). Negative controls consisted of RT reactions without template. Samples were separated on 1.2% agarose gels and visualized with ethidium bromide using a Syngene GBox.

**Table 1.** Primer sequences

Target	Primer	Sequence	Size (bp)
Exon 1–3	Exon 1 F	5′-GAGGCAGCAGCATACCAGAA-3′	753
Exon 3 R	5′-TTTCCGGAGACGACACGATG-3′
Exon 2–3	Exon 2 F	5′-CAGGGACCACGTTTTACCCA-3′	229
Exon 3 R	5′-TTTCCGGAGACGACACGATG-3′
Exon 3–4	Exon 3 R	5′-GACTCTGGGAGCTCGTAAGC-3′	185
Exon 4 R	5′-ACACCACTCCTGGCTCAATG-3′
Exon 4–5	Exon 4 F	5′-GTGCCGGACATGACAACAAC-3′	281
Exon 5 R	5′-TCGAGACTTGTGCATGCGAT-3′
Exon 6–7	Exon 6 F	5′-AATGTACAGCCAGTGCGTGA-3′	248
Exon 7 R	5′-TTGGTGAGCTGGTAGAAGCG-3′

### Bioassays

During the first 2 h of the circadian light phase, each rat was anesthetized with 2.5% isoflurane and decapitated. Prostate and seminal vesicle wet tissue weights were measured. Trunk blood (5–7 ml) was obtained in EDTA-coated tubes (13 × 100 mm, Covidien) on ice. The blood was centrifuged (Allegra X-30R, Beckman Coulter) at 2000 × *g* for 10 min at 4°C, then separated plasma was placed in microcentrifuge tubes and stored at –80°C until ELISA analysis for total testosterone. Plasma testosterone levels were assayed according to manufacturer’s instructions. The intra-assay coefficient of variation was 8.54%, and the interassay coefficient of variation was 9.97%. The sensitivity of this assay was 0.066 ng/ml testosterone. The specificity of this assay is 100% testosterone, 69.6% dihydrotestosterone (DHT), 7.4% dihydroxyandrosterone, and <0.1% for androstenedione, androsterone, epiandrosterone, dihydroandrosterone, estrone, estradiol, estriol, cortisol, 11-deoxycortisol, progesterone, and 17OH-progesterone.

### Statistical analysis

Analysis was performed using IBM SPSS Statistics, version 21. Data were expressed as mean ±SEM. Significance (*p* ≤ 0.05) was determined by ANOVA.

## Results

### Bioassays

Body weights, prostate weights, seminal vesicle weights, and total testosterone levels were quantified (Table 2). Hormone treatment did not have a significant impact on body weight, regardless of age. However, middle-aged rats (9–12 mo) were significantly heavier than young rats (3 mo; *F*_1,45_ = 233.284, *p* < 0.05). One week after gonadectomy (GDX), plasma testosterone levels were significantly decreased compared with gonadally intact rats, regardless of age (*F*_1,45_ = 54.221, *p* < 0.05). No differences in testosterone levels between young and middle-aged gonadally intact rats were observed. Consistent with a decline in testosterone, GDX rats exhibited a significant decrease in weights for androgen-sensitive accessory organs: prostate (*F*_1,45_ = 74.117, *p* < 0.05) and seminal vesicles (*F*_1,45_ = 33.088, *p* < 0.05). Middle-aged retired breeders, regardless of hormone condition, had significantly heavier prostate (*F*_1,45_ = 23.577, *p* < 0.05) and seminal vesicle (*F*_1,45_ = 42.929, *p* < 0.05) weights than young sexually naive rats. These results are consistent with prior studies showing that sexual experience increases testosterone and androgen-sensitive accessory organ weights (Drori and Folman, 1964; Thomas and Neiman, 1968; Aumüller et al., 1985; Cummings et al., 2013).

**Table 2.** Bioassays

Age	Hormone	*n*	Body weight	Prostate	Seminal vesicles	Total testosterone
Young	GDX	13	325.25 ± 7.75	0.16 ± 0.08^#^	0.40 ± 0.18^#^	0.36 ± 0.63^#^
Young	Intact	13	325.35 ± 27.75	0.46 ± 0.13	0.83 ± 0.19	5.61 ± 2.90
Middle-aged	GDX	12	444.75 ± 26.48*	0.30 ± 0.11*^#^	0.89 ± 0.20*^#^	0.21 ± 0.18^#^
Middle-aged	Intact	11	456.68 ± 43.96*	0.75 ± 0.25*	1.27 ± 0.39*	4.34 ± 3.42

Young rats are 3 mo old; middle-aged rats are 9–12 mo old. Weights are expressed as mean g ± SEM. Plasma testosterone levels are expressed as mean ng/ml ± SEM. GDX, gonadectomy; intact, gonadally intact. *P* < 0.05: *versus young; ^#^versus intact.

### Androgen receptor expression

Androgen receptor expression was quantified in a dopaminergic N27 cell line, along with brain tissue from the substantia nigra pars compacta (SN), second layer of the entorhinal cortex (ETC), and CA1 layer of the dorsal hippocampus from young and middle-aged male rats that were either gonadally intact or GDX (Fig. 1). Frozen and fresh tissue were used, since prior reports found that low temperatures can increase full-length AR fragments (Kemppainen et al., 1992; Gregory et al., 2001). Antibodies targeting either the NTD or CTD of the AR were used to examine AR protein expression. Protein from testes was used as a positive control for AR expression. Low protein expression of full-length AR (110 kDa) was found in all samples using both NTD (AR N20) and CTD (AR C-19) AR antibodies. High protein expression of AR fragments (70 and 30 kDa) were observed in fresh samples and to a lesser extent in frozen samples (Fig. 1*A*). These likely represent calpain-dependent proteolysis, as described by Pelley et al. (2006), in human prostate cancer cells. Protein expression at 45 kDa was observed in all samples and was unaffected by temperature. Further, neither hormone nor age altered AR45 protein expression in N27 cells, SN tissue, and testes (Fig. 1*B*, *C*). Interestingly, this 45-kDa band was evident only when using a CTD-targeted antibody for the AR and not an NTD antibody, consistent with the AR splice variant AR45 that lacks an NTD. Similar results were observed using different antibodies (AR441 and R/NR3C4) targeting the NTD and CTD of the AR (data not shown).

**Figure 2.**
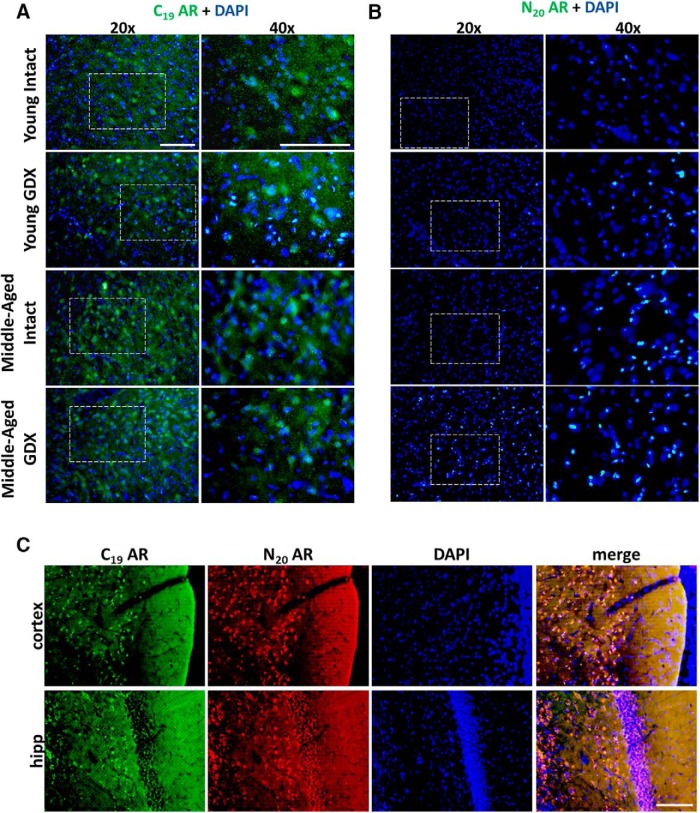
Widespread AR45 distribution in the substantia nigra. AR immunoreactivity using a CTD targeted antibody (AR C19) was observed throughout the SN in all age and hormone groups (***A***). No AR immunoreactivity was observed using a NTD targeted antibody in the SN (***B***). Both the hippocampus and the ETC express CTD and NTD AR immunoreactivity that is present within the nucleus, as evidenced by DAPI colocalization (***C***). SN, substantia nigra pars compacta; ETC, 2nd layer of the entorhinal cortex; Hipp, CA1 region of the dorsal hippocampus. Scale bar = 200 μm.

Widespread CTD AR immunoreactivity (and not NTD AR–positive cells) was observed throughout the SN in young and middle-aged rats, respective of hormone and age status (Fig. 2*A*, *B*). AR immunoreactivity included extranuclear staining, suggesting membrane or cytosolic localization. In contrast, the hippocampus and the ETC express both CTD- and NTD-positive AR within the nucleus in young intact males (Fig. 2*C*), consistent with prior immunohistochemical studies using AR antibodies (Sar et al., 1990; Xiao and Jordan, 2002; DonCarlos et al., 2003; Kritzer, 2004).

### Expression profile of androgen receptors

**Figure 3.**
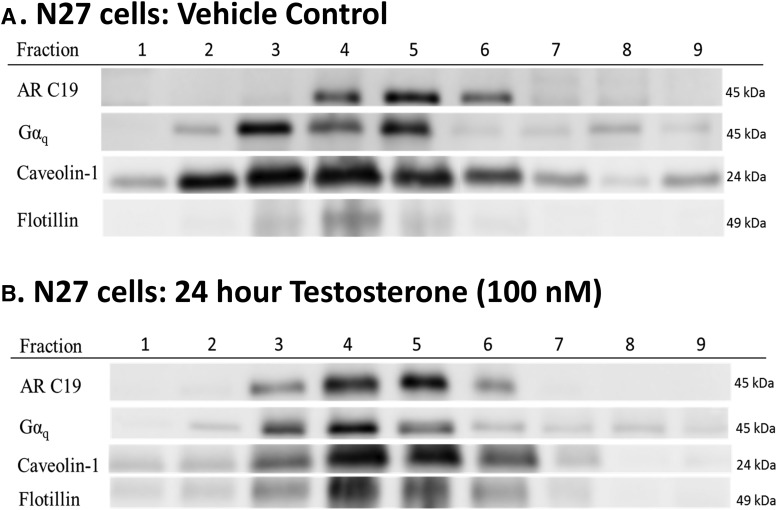
N27 cells express AR45 protein in membrane lipid rafts. N27 cells were treated with either vehicle control (***A***) or 100 nM testosterone for 24 h (***B***). The membrane portion of the cells were further separated into nine fractions using a sucrose gradient and ultracentrifugation to examine lipid rafts. Primary antibodies targeting AR45 (AR-C19 antibody), G protein Gαq, and lipid raft markers (caveolin-1 and flotillin) were used. AR45 and Gαq expression were observed only in lipid raft fractions, as evidenced by caveolin-1 and flotillin expression. Hormone treatment did not alter AR45 and Gαq expression in lipid rafts. Full-length (110-kDa) AR, Gαo, Gαi_1-3_, and Gαs G proteins were not observed in the membranes of N27 cells (data not shown). *n* = 3 per treatment group.

**Figure 4.**
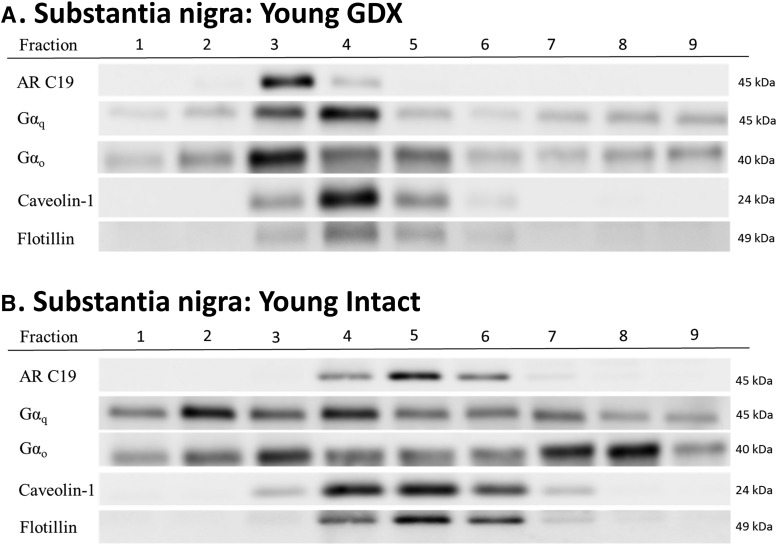
Young male rats express AR45 protein in membrane lipid rafts. Young rats (3 mo old) were either gonadectomized (***A***) or gonadally intact (***B***). Micropunches of substantia nigra tissue were collected, and the membrane was isolated and then separated into nine fractions using a sucrose gradient to examine lipid rafts. Primary antibodies targeting AR45 (AR-C19 antibody), G proteins Gαq and Gαo, and lipid raft markers (caveolin-1 and flotillin) were used. AR45 expression was observed only in lipid raft fractions, as evidenced by caveolin-1 and flotillin expression. Hormone status did not alter AR45 expression in lipid rafts. Gαq and Gαo was observed throughout the membrane, regardless of hormone status. No protein expression of Gαi_1-3_ and Gαs G proteins or full-length (100-kDa) AR was observed in the substantia nigral membranes of young male rats (data not shown). GDX, gonadectomy; intact, gonadally intact. *n* = 3 per treatment group.

**Figure 5.**
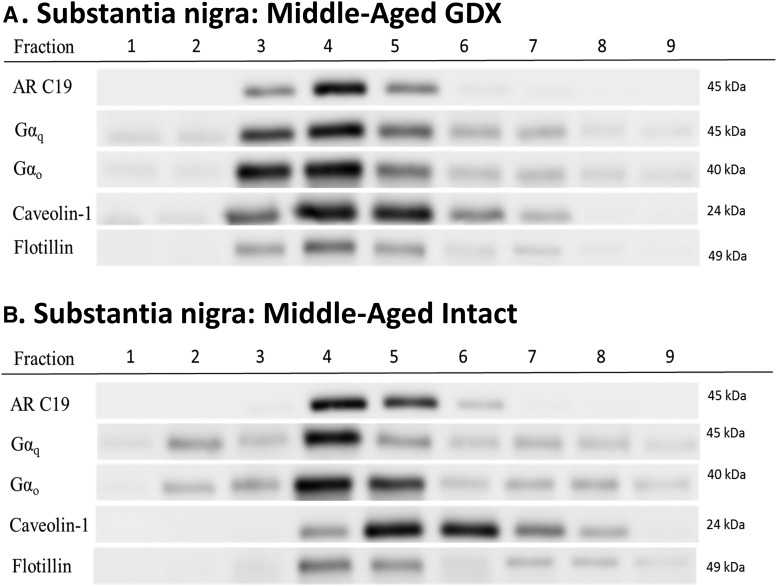
Aged male rats express AR45 protein in membrane lipid rafts. Middle-aged rats (9- to 12-mo old retired breeders) were either gonadectomized (***A***) or gonadally intact (***B***). Membrane portion of the substantia nigra micropunch tissue was isolated, and then separated into nine fractions using a sucrose gradient. AR45 (AR-C19 antibody), Gαq and Gαo, and lipid raft markers (caveolin-1 and flotillin) were examined. AR45 expression was observed only in lipid raft fractions, regardless of hormone status. Gαq and Gαo was observed throughout the membrane in both gonadectomized and gonadally intact males. Protein expression of Gαi_1-3_, Gαs, or full-length (110-kDa) AR were not observed in the substantia nigral membranes of aged male rats (data not shown). GDX, gonadectomy; intact, gonadally intact. *n* = 3 per treatment group.

N27 cells and SN brain tissue were split into membrane, cytosol, and nuclear fractions. The membrane portion of both N27 cells and SN brain tissue was further separated into nine fractions using a sucrose gradient (Figs. 3–5). Full-length AR at 110 kDa was not observed in any membrane fraction. Although full-length AR was not expressed, a 45-kDa protein corresponding to AR45 was observed in all samples. In N27 cells, regardless of testosterone exposure, AR45 expression was evident in fractions 3–6, which are lipid rafts as shown by caveolin-1 and flotillin immunoreactivity (Fig. 3*A*, *B*). Similarly, AR45 immunoreactivity was present in caveolin- and flotillin-enriched membrane lipid rafts in both young and middle-aged rats in all hormone groups (Figs. 4–5). AR45 immunoreactivity was not found in any non–lipid raft portion of the membrane, nor was it observed in cytosolic or nuclear fractions.

### Androgen receptor mRNA expression

**Figure 6.**
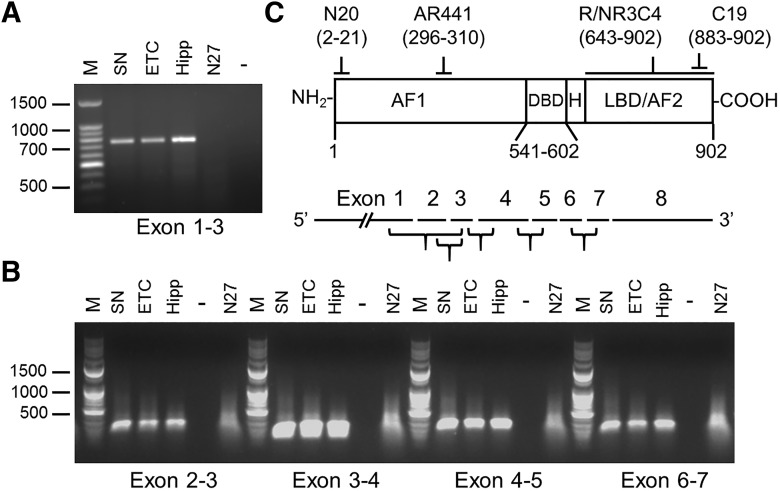
Androgen receptor RT-PCR. Intron-spanning primer sets for rat AR were used to amplify cDNA from young intact male rat substantia nigra pars compacta (SN), 2nd layer of the entorhinal cortex (ETC), CA1 region of the hippocampus (Hip), and N27 cells (***A*** and ***B***). Note the lack of amplification product for the 3′ region of AR in N27 cells (***A***). ***C***, Targets of the antibodies used to detect AR and their epitopes in parentheses aligned with the AR domain structure (top), and the relative amplification product locations in the AR mRNA (bottom). Exons 2 and 3 code for the DBD. M denotes a 100-bp DNA ladder, and negative controls are marked with –.

RT-PCR for AR was performed to determine whether full-length transcripts were present in SN and N27 cells (Fig. 6). Several intron-spanning primer sets were used to amplify equivalent amounts of cDNA under identical conditions. In the brain, we detected positive signals from all regions examined (SN, ETC, Hipp), confirming the presence of full-length AR mRNA (Fig. 6*A*, *B*). However, in N27 cells, we detected no product from an exon 1 to exon 3 primer pair. Exon 2–7 amplicons were detected in N27 cells (Fig. 6*B*), although the signal was less distinct than in brain samples. These data suggest that full-length AR mRNA is not present in N27 cells, or is present only at very low levels, below detectability under our conditions (35 cycles, EtBr detection).

### Expression profile of GPCRs in membrane lipid rafts

Because neuronal mAR has been associated with intracellular calcium signaling (Steinsapir et al., 1991; Gorczynska and Handelsman, 1995; Benten et al., 1999; Estrada et al., 2003, 2006; Holmes et al., 2013), we examined whether G proteins were present in lipid rafts. In both N27 cells and SN tissue, the G protein Gαq was expressed in the membrane fraction and in lipid rafts, but Gαi_1-3_ and Gαs were not expressed in any of the membrane fractions. Interestingly, the G protein Gαo was expressed only in SN tissue and not in the N27 cell line (Figs. 3–5).

### Androgen receptor variant association with GPCR subunits

**Figure 7.**
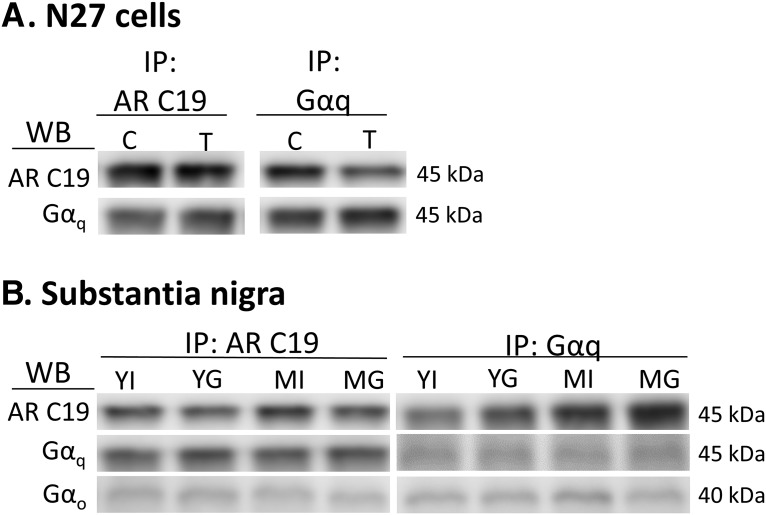
Coimmunoprecipitation (IP) of the AR45-G protein complex. Whole-cell lysates of N27 cells treated with either vehicle or 100 nM testosterone (***A***) and substantia nigral tissue from young and middle-aged male rats that were either gonadectomized or gonadally intact (***B***) were used. AR CTD-containing AR45 proteins were immunoprecipitated and then immunoblotted (WB) for Gαq and Gαo. In a reciprocal fashion, Gαq-containing proteins were immunoprecipitated and then immunoblotted for AR45. In N27 cells, bands corresponding to AR45 (AR-C19) and Gαq were detectable. In substantia nigral tissue, bands corresponding to AR45 (AR-C19), Gαq, and Gαo were observed. C, control; T, testosterone; YI, young gonadally intact male rats; YG, young GDX male rats; MI, middle-aged gonadally intact male rats; MG, middle-aged GDX male rats. *n* = 4 per treatment group.

To determine whether AR45 interacts with G proteins, coimmunoprecipitation was performed. Antibodies targeting the CTD of the AR were used to pull down proteins. In a reciprocal fashion to ensure specificity, Gαq-containing proteins were immunoprecipitated and then immunoblotted for AR45 with an AR C19 antibody. After electrophoresis, membranes were probed for AR45 (C-19 antibody) or G protein immunoreactivity. In N27 cells, AR45 interacted with Gαq, regardless of testosterone exposure (Fig. 7*A*). Furthermore, in SN tissue, AR45 was associated with Gαq and Gαo in all hormone states and ages (Fig. 7*B*).

## Discussion

This study found AR protein expression at 45-kDa molecular weight in all samples examined: N27 cells, SNpc, hippocampus, ETC, and testes. Furthermore, this AR variant is present in plasma membrane lipid rafts from N27 cells and SN brain tissue from young and middle-aged rats with and without sex hormones. The 45-kDa membrane protein was evident only using a CTD-targeted antibody for AR, consistent with the AR45 splice variant. Interestingly, protein expression of AR45 was not altered by steroid hormones, age, or temperature. Although full-length AR mRNA was found in all brain samples examined, little to no AR immunoreactivity using the NTD-targeted AR antibody was observed in SNpc tissue, unlike the ETC and hippocampus. Expression profiles for AR immunoreactivity for these brain regions are different, indicating different mechanisms of androgen action in SN versus hippocampus and ETC. This result, coupled with a lack of a detectable exon 1–containing transcript in N27 cells, suggests that the immunoreactive AR45 may represent a bona fide splice variant rather than posttranslational processing of the full-length AR.

Prior studies in young adult and adolescent male Sprague-Dawley rats described AR immunoreactivity in the SNpc using AR antibodies targeting the NTD of the receptor (Kritzer, 1997; Kritzer and Creutz, 2008; Purves-Tyson et al., 2012). Our results are consistent with the Kritzer laboratory’s findings of low NTD-AR immunoreactivity within the SNpc (only the dorsomedial region) but no AR expression in the rest of the SNpc obtained from young adult male rats. The highest AR immunoreactivity was observed in SN pars lateralis, wherein >50% of the neurons are AR^+^ (Kritzer, 1997; Kritzer and Creutz, 2008). These findings indicate that full-length AR is not highly expressed in the SNpc of young adult male rats. In contrast, adolescent male rats have high NTD-AR immunoreactivity in the SNpc, as evidenced by >65% of the TH^+^ neurons immunoreactive for AR (Purves-Tyson et al., 2012). This difference in NTD-AR immunoreactivity between young adult and adolescent male rats could be due to adolescent pruning of TH^+^ neurons in the SNpc (Kuhn et al., 2010). To the best of the authors’ knowledge, there are no prior immunohistochemical studies using CTD-AR antibodies in the SN.

Because classic full-length AR protein was not highly present in SNpc and N27 cells, it is possible that the AR45 splice variant is mediating androgen’s actions in this brain region by acting as an mAR. Prior studies, using nonneuronal cells, have linked AR to membrane lipid rafts (Lu et al., 2001; Cinar et al., 2007; Pedram et al., 2007). Lipid rafts are low-density microdomains, enriched with cholesterol and lipids, and insoluble in non-ionic detergents (Brown and Rose, 1992; Brown and London, 2000). The proteins flotillin and caveolin are integral components of lipid rafts. Neuronal lipid rafts generally are planar and composed of flotillin, unlike nonneuronal cells that contain rafts with invaginations composed of caveolin (Murata et al., 1995; Bickel et al., 1997; Lang et al., 1998). However, studies have shown that caveolin-1 can be present in neurons under certain conditions, such as oxidative stress and aging (Volonté et al., 2001; Kang et al., 2006; Marquet-de Rougé et al., 2013). One of the brain regions with the highest expression of caveolin-1 is the SN (Galbiati et al., 1998), which is composed mainly of dopaminergic TH^+^ neurons that have increased oxidative stress from dopamine metabolism (Lotharius et al., 2002; Hastings, 2009).

Our data showed that AR45 is present in membrane fractions from N27 cells and SN tissue. Specifically, AR45 was present only in caveolin- and flotillin-enriched lipid rafts, indicating localization to lipid rafts that contain invaginations. Although AR45 was expressed only in caveolin^+^ lipid rafts, differences were observed in which membrane fractions these proteins localized to. Caveolin was more widespread in the membrane fractions from the N27 cell line, wherein caveolin were observed in lower-density protein fractions. Notably, testosterone appeared to shift caveolin expression to higher-density protein fractions in both N27 cells and SN tissue. It is possible that caveolin is undergoing posttranslational modification, resulting in higher-density proteins. Posttranslational modifications (e.g., palmitoylation and phosphorylation) commonly occur in caveolin proteins and steroid receptors (Parat and Fox, 2001; Fukata and Fukata, 2010; Kim et al., 2011). A common initiator of posttranslational modifications in caveolin includes oxidative stress (Kim et al., 2000; Volonté et al., 2001; Wehinger et al., 2015), and testosterone can act as an oxidative stressor (Holmes et al., 2013, 2016). Therefore, it is possible that testosterone, via oxidative stress, is increasing posttranslational modifications in caveolin protein, resulting in the caveolin/AR45 complex shifting to higher-density protein fractions.

Numerous signaling proteins, such as receptor tyrosine kinases, GPCRs, and G proteins, reside in lipid rafts and play a pivotal role in signal transduction (Mineo et al., 1999; Toki et al., 1999; Brown and London, 2000; Lasley et al., 2000; Rybin et al., 2000; Rebois and Hébert, 2003; Pucadyil and Chattopadhyay, 2004; Allen et al., 2005; Xu et al., 2006). Prior studies have shown that Gαq mainly localizes in caveolae lipid rafts, unlike Gαs and Gαi proteins (Murthy and Makhlouf, 2000; Oh and Schnitzer, 2001; Sengupta et al., 2008). Indeed, the results from this study show that Gαq and Gαo proteins are present in lipid rafts, along with AR45. Furthermore, AR45 coimmunoprecipitates with Gαq and Gαo proteins, indicating that AR45 interacts with G proteins in lipid rafts in dopaminergic cells.

Gαq has been well established as an activator of intracellular calcium release from the endoplasmic reticulum (Rhee, 2001; Shi and Kehrl, 2001), which can affect dopaminergic neuronal function in the SNpc (Surmeier et al., 2012). Much less is known about the function of Gαo proteins that are highly present in frontal cortex, cerebellum, hypothalamus, hippocampus, and SN (Worley et al., 1986). Gαo can couple to receptors that decrease intracellular calcium release (Lewis et al., 1986; Hescheler et al., 1987), and in the brain Gαo is predominantly coupled to inhibitory D2 dopamine receptors (Jiang et al., 2001). Interestingly, this association with D2 receptors may explain the lack of Gαo expression in the N27 cells, as D2 receptors are not expressed in this cell line (Urban and Mailman, 2005). Further supporting the role of Gαo in SN dopaminergic neuronal involvement, Gαo knockout mice exhibit poor motor coordination (Jiang et al., 1998). Although data about Gαo is sparse, Gαo is linked with motor function and calcium signaling.

Because AR45 appears to be the predominant AR in the SNpc, it may have clinical relevance in the progressive motor disorder Parkinson’s disease (PD), resulting from the loss of TH^+^ neurons in the SNpc (Pike et al., 2006, 2009; Cunningham et al., 2009, 2011; Rosario et al., 2011; Holmes et al., 2013, 2016; Verdile et al., 2014). Men have a twofold increased risk for PD than women (Baldereschi et al., 2000). Similarly, postmenopausal women have a greater risk for PD than age-matched premenopausal women (Currie et al., 2004; Ragonese et al., 2006a, 2006b), which may be due to the higher circulating androgen-to-estrogen state during menopause (Vermeulen, 1976; Laughlin et al., 2000; Liu et al., 2001; Handelsman et al., 2016; Labandeira-Garcia et al., 2016). It is unknown what mechanisms underlie this PD sex difference, but it is possible that AR45 located in dopaminergic membrane caveolin lipid rafts may be involved, especially as upregulated caveolin expression and lipid rafts have been linked with PD (Hashimoto et al., 2003; Trushina et al., 2006; Schengrund, 2010; Sonnino et al., 2014; Cha et al., 2015). Furthermore, recent *in vitro* studies support the involvement of an mAR in mediating cell viability, oxidative stress generation, and calcium signaling in dopaminergic cells (Estrada et al., 2006; Holmes et al., 2013, 2016), which are key characteristics and processes observed in PD pathology (Jenner et al., 1992; Sherer et al., 2002; Dickson, 2007). Interestingly, both of the *in vitro* cell lines (N27 and SH-SY5Y cells) are female-derived cell lines, indicating that these fast, nongenomic androgen actions in neurons are (1) applicable to both males and females and (2) dependent on the hormonal and AR expression milieu.

**Figure 8.**
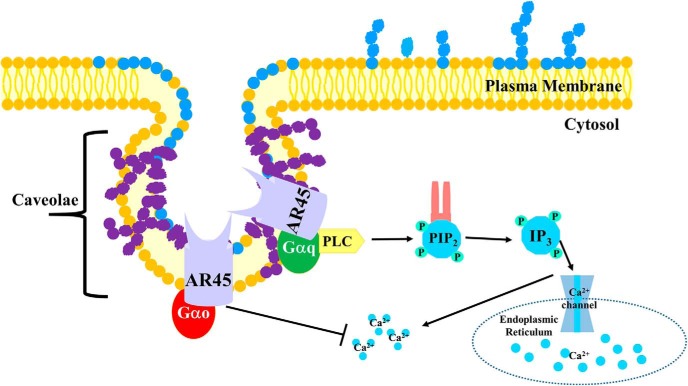
Model. Caveolae lipid rafts are flask-shaped invaginations made up of caveolin (purple) and flotillin (blue) proteins that function by organizing signaling complexes in the plasma membrane. Proteins present in caveolae include AR45. Furthermore, AR45 proteins interact with Gαq and Gαo proteins, which are involved in modulating intracellular calcium levels. Gαq can increase intracellular calcium release via the PLC/IP3 pathway, whereas Gαo can inhibit intracellular calcium. PLC, phospholipase C, IP3: inositol trisphosphate.

This is the first study to show the presence of a putative AR splice variant protein in the SNpc, hippocampus, and ETC brain regions. Specifically, our results show that AR45 localizes in the membrane lipid rafts from N27 cells and SN dopaminergic neurons. Furthermore, AR45 interacts with Gαq and Gαo G proteins, which can impact intracellular calcium signaling (Fig. 8). More research needs to be conducted to further determine the function and role of this AR splice variant in dopaminergic neuronal function and dysfunction.

## Synthesis

Reviewing Editor: Jeffrey Blaustein, University of Massachusetts Decisions are customarily a result of the Reviewing Editor and the peer reviewers coming together and discussing their recommendations until a consensus is reached. When revisions are invited, a fact-based synthesis statement explaining their decision and outlining what is needed to prepare a revision will be listed below. The following reviewer(s) agreed to reveal their identity: Toni Pak

Although I am accepting as is, when you get the proofs, please make the following corrections:

1. line 245, identify syngene Gbox.

2. line 259: estrone misspelled

I think this will be more expedient than sending it back to you now.
